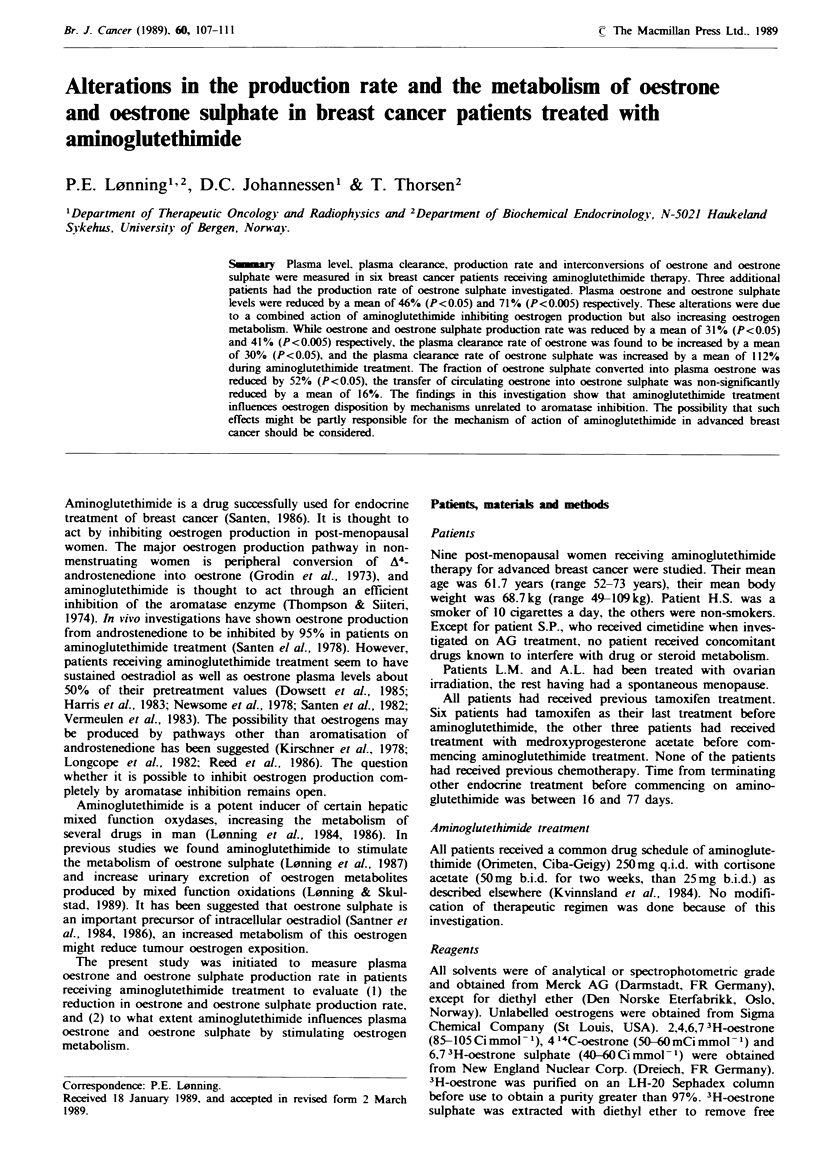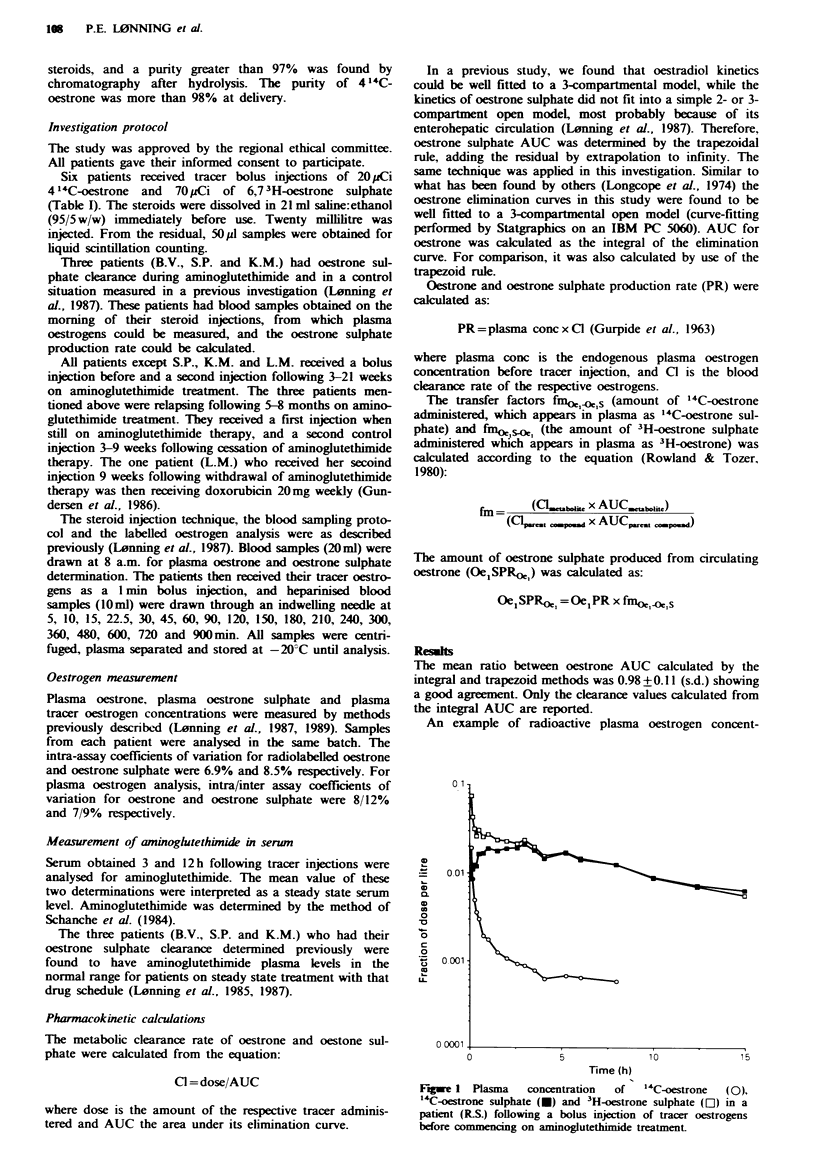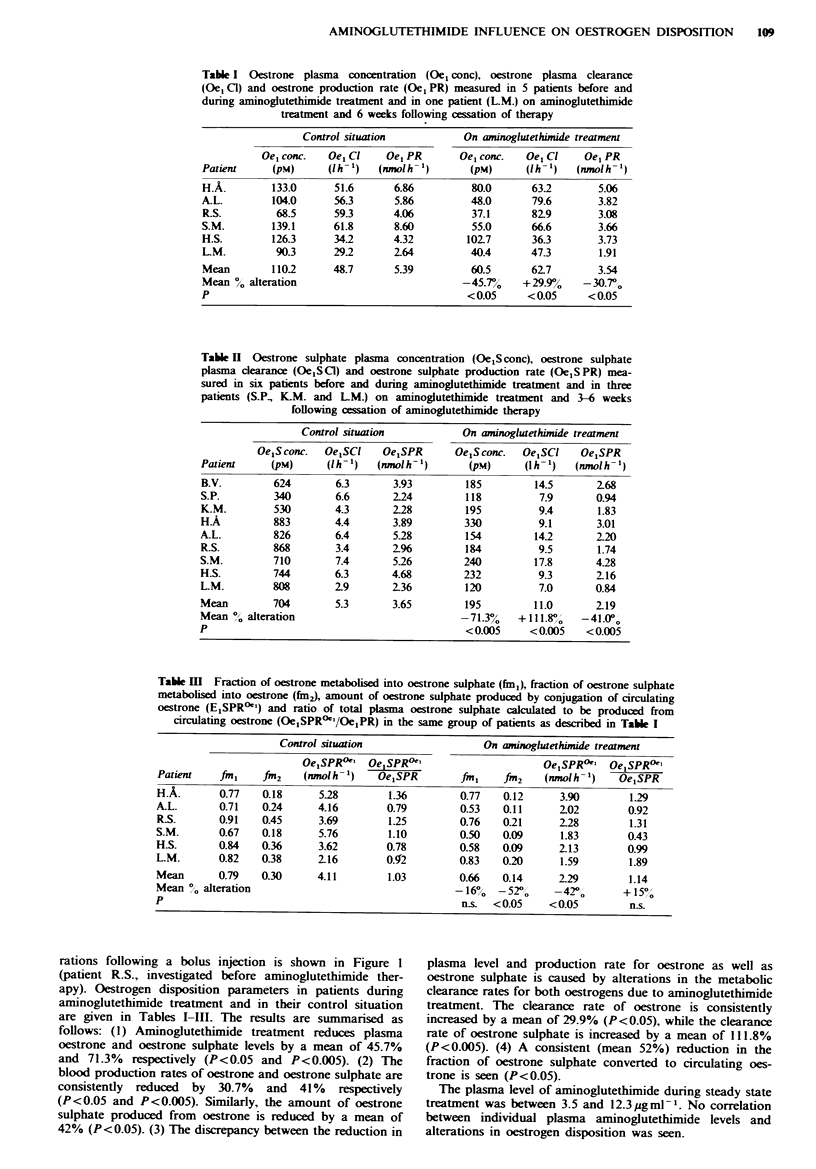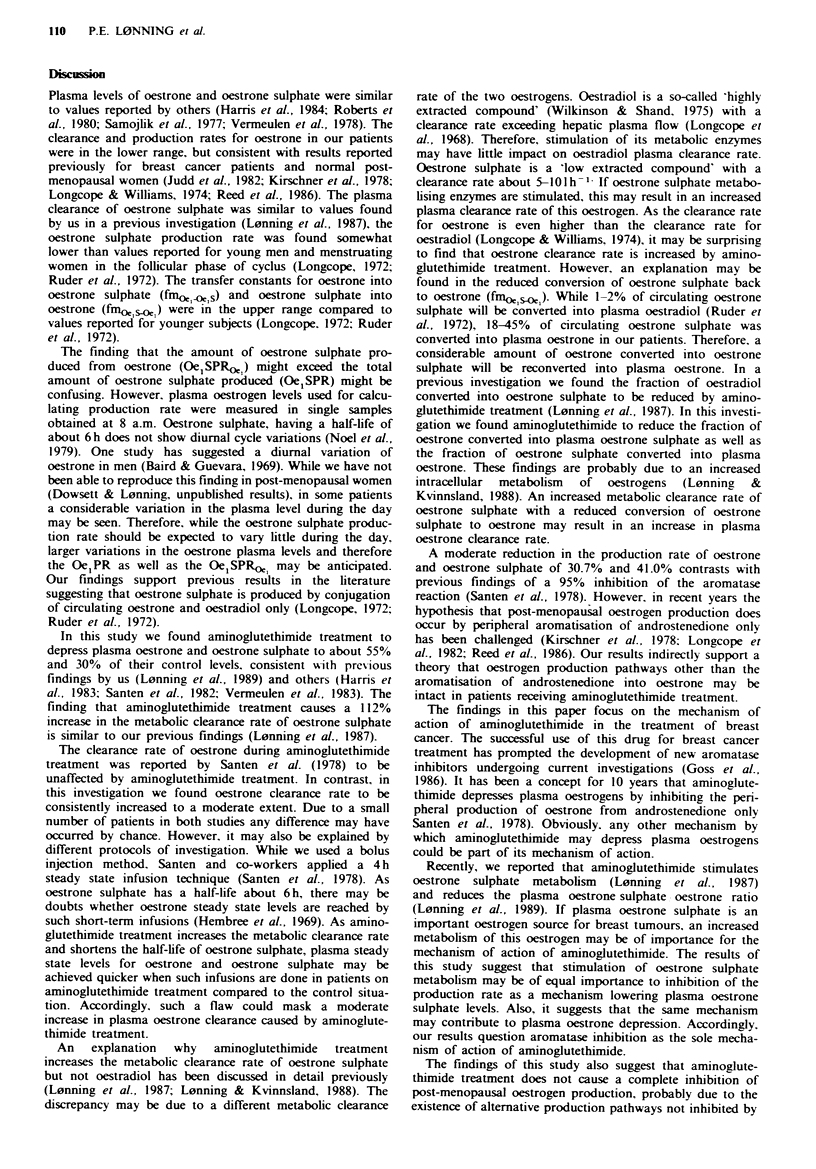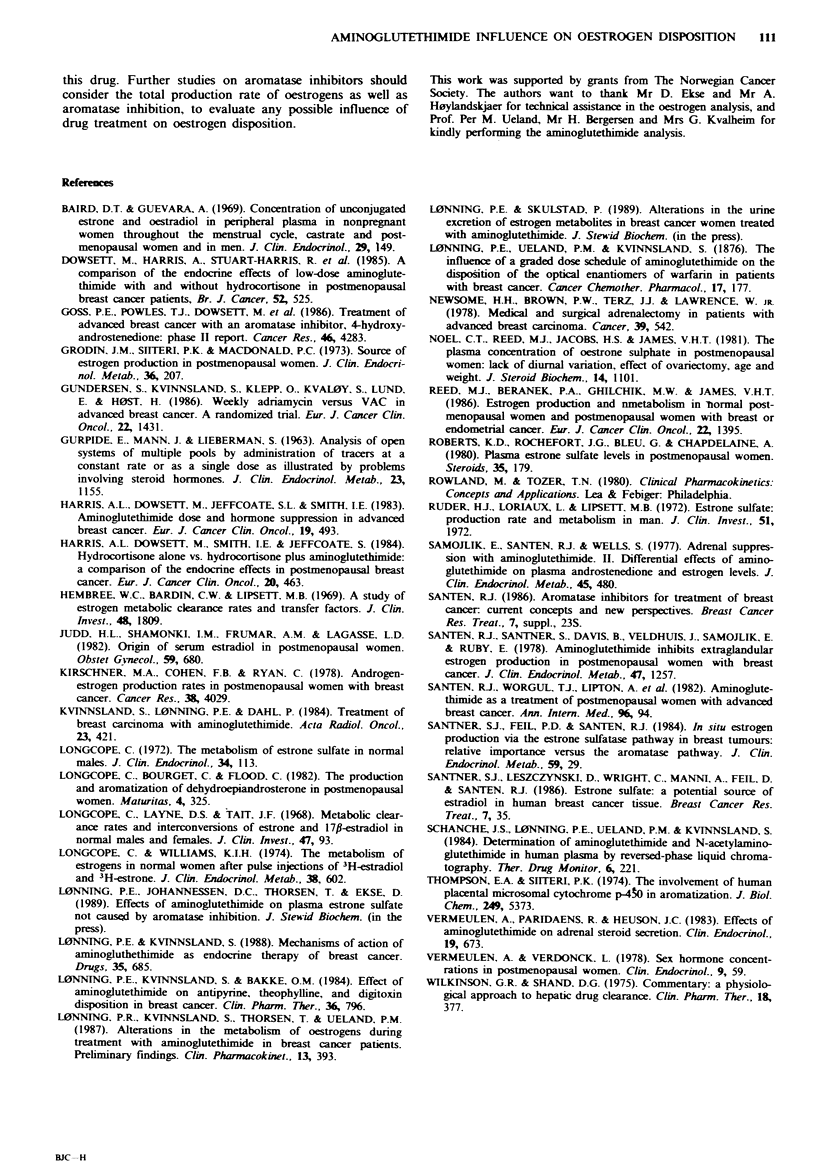# Alterations in the production rate and the metabolism of oestrone and oestrone sulphate in breast cancer patients treated with aminoglutethimide.

**DOI:** 10.1038/bjc.1989.231

**Published:** 1989-07

**Authors:** P. E. LÃ¸nning, D. C. Johannessen, T. Thorsen

**Affiliations:** Department of Therapeutic Oncology and Radiophysics, University of Bergen, Norway.

## Abstract

Plasma level, plasma clearance, production rate and interconversions of oestrone and oestrone sulphate were measured in six breast cancer patients receiving aminoglutethimide therapy. Three additional patients had the production rate of oestrone sulphate investigated. Plasma oestrone and oestrone sulphate levels were reduced by a mean of 46% (P less than 0.05) and 71% (P less than 0.005) respectively. These alterations were due to a combined action of aminoglutethimide inhibiting oestrogen production but also increasing oestrogen metabolism. While oestrone and oestrone sulphate production rate was reduced by a mean of 31% (P less than 0.05) and 41% (P less than 0.005) respectively, the plasma clearance rate of oestrone was found to be increased by a mean of 30% (P less than 0.05), and the plasma clearance rate of oestrone sulphate was increased by a mean of 112% during aminoglutethimide treatment. The fraction of oestrone sulphate converted into plasma oestrone was reduced by 52% (P less than 0.05), the transfer of circulating oestrone into sulphate was non-significantly reduced by a mean of 16%. The findings in this investigation show that aminoglutethimide treatment influences oestrogen disposition by mechanisms unrelated to aromatase inhibition. The possibility that such effects might be partly responsible for the mechanism of action of aminoglutethimide in advanced breast cancer should be considered.


Br. J. Cancer (1989), 60, 107–111 © The Macmillan Press Ltd., 1989

### Alterations in the production rate and the metabolism of oestrone and oestrone sulphate in breast cancer patients treated with aminoglutethimide

P.E. Lønning[1,2], D.C. Johannessen[1] & T. Thorsen[2]

[1]*Department of Therapeutic Oncology and Radiophysics and* [2]*Department of Biochemical Endocrinology, N-5021 Haukeland Sykehus, University of Bergen, Norway.*

**Summary** Plasma level, plasma clearance, production rate and interconversions of oestrone and oestrone sulphate were measured in six breast cancer patients receiving aminoglutethimide therapy. Three additional patients had the production rate of oestrone sulphate investigated. Plasma oestrone and oestrone sulphate levels were reduced by a mean of 46% ($P<0.05$) and 71% ($P<0.005$) respectively. These alterations were due to a combined action of aminoglutethimide inhibiting oestrogen production but also increasing oestrogen metabolism. While oestrone and oestrone sulphate production rate was reduced by a mean of 31% ($P<0.05$) and 41% ($P<0.005$) respectively, the plasma clearance rate of oestrone was found to be increased by a mean of 30% ($P<0.05$), and the plasma clearance rate of oestrone sulphate was increased by a mean of 112% during aminoglutethimide treatment. The fraction of oestrone sulphate converted into plasma oestrone was reduced by 52% ($P<0.05$), the transfer of circulating oestrone into oestrone sulphate was non-significantly reduced by a mean of 16%. The findings in this investigation show that aminoglutethimide treatment influences oestrogen disposition by mechanisms unrelated to aromatase inhibition. The possibility that such effects might be partly responsible for the mechanism of action of aminoglutethimide in advanced breast cancer should be considered.

Aminoglutethimide is a drug successfully used for endocrine treatment of breast cancer (Santen, 1986). It is thought to act by inhibiting oestrogen production in post-menopausal women. The major oestrogen production pathway in non-menstruating women is peripheral conversion of $\Delta^4$-androstenedione into oestrone (Grodin *et al.*, 1973), and aminoglutethimide is thought to act through an efficient inhibition of the aromatase enzyme (Thompson & Siiteri, 1974). *In vivo* investigations have shown oestrone production from androstenedione to be inhibited by 95% in patients on aminoglutethimide treatment (Santen *el al.*, 1978). However, patients receiving aminoglutethimide treatment seem to have sustained oestradiol as well as oestrone plasma levels about 50% of their pretreatment values (Dowsett *et al.*, 1985; Harris *et al.*, 1983; Newsome *et al.*, 1978; Santen *et al.*, 1982; Vermeulen *et al.*, 1983). The possibility that oestrogens may be produced by pathways other than aromatisation of androstenedione has been suggested (Kirschner *et al.*, 1978; Longcope *et al.*, 1982; Reed *et al.*, 1986). The question whether it is possible to inhibit oestrogen production completely by aromatase inhibition remains open.

Aminoglutethimide is a potent inducer of certain hepatic mixed function oxydases, increasing the metabolism of several drugs in man (Lønning *et al.*, 1984, 1986). In previous studies we found aminoglutethimide to stimulate the metabolism of oestrone sulphate (Lønning *et al.*, 1987) and increase urinary excretion of oestrogen metabolites produced by mixed function oxidations (Lønning & Skulstad, 1989). It has been suggested that oestrone sulphate is an important precursor of intracellular oestradiol (Santner *et al.*, 1984, 1986), an increased metabolism of this oestrogen might reduce tumour oestrogen exposition.

The present study was initiated to measure plasma oestrone and oestrone sulphate production rate in patients receiving aminoglutethimide treatment to evaluate (1) the reduction in oestrone and oestrone sulphate production rate, and (2) to what extent aminoglutethimide influences plasma oestrone and oestrone sulphate by stimulating oestrogen metabolism.

Correspondence: P.E. Lønning.
Received 18 January 1989, and accepted in revised form 2 March 1989.

### Patients, materials and methods

### *Patients*

Nine post-menopausal women receiving aminoglutethimide therapy for advanced breast cancer were studied. Their mean age was 61.7 years (range 52–73 years), their mean body weight was 68.7 kg (range 49–109 kg). Patient H.S. was a smoker of 10 cigarettes a day, the others were non-smokers. Except for patient S.P., who received cimetidine when investigated on AG treatment, no patient received concomitant drugs known to interfere with drug or steroid metabolism.

Patients L.M. and A.L. had been treated with ovarian irradiation, the rest having had a spontaneous menopause.

All patients had received previous tamoxifen treatment. Six patients had tamoxifen as their last treatment before aminoglutethimide, the other three patients had received treatment with medroxyprogesterone acetate before commencing aminoglutethimide treatment. None of the patients had received previous chemotherapy. Time from terminating other endocrine treatment before commencing on aminoglutethimide was between 16 and 77 days.

### *Aminoglutethimide treatment*

All patients received a common drug schedule of aminoglutethimide (Orimeten, Ciba-Geigy) 250 mg q.i.d. with cortisone acetate (50 mg b.i.d. for two weeks, than 25 mg b.i.d.) as described elsewhere (Kvinnsland *et al.*, 1984). No modification of therapeutic regimen was done because of this investigation.

### *Reagents*

All solvents were of analytical or spectrophotometric grade and obtained from Merck AG (Darmstadt, FR Germany), except for diethyl ether (Den Norske Eterfabrikk, Oslo, Norway). Unlabelled oestrogens were obtained from Sigma Chemical Company (St Louis, USA). 2,4,6,7 $^3$H-oestrone (85–105 Ci mmol$^{-1}$), 4 $^{14}$C-oestrone (50–60 mCi mmol$^{-1}$) and 6,7 $^3$H-oestrone sulphate (40–60 Ci mmol$^{-1}$) were obtained from New England Nuclear Corp. (Dreiech, FR Germany). $^3$H-oestrone was purified on an LH-20 Sephadex column before use to obtain a purity greater than 97%. $^3$H-oestrone sulphate was extracted with diethyl ether to remove free steroids, and a purity greater than 97% was found by chromatography after hydrolysis. The purity of 4 $^{14}$C-oestrone was more than 98% at delivery.

*Investigation protocol*

The study was approved by the regional ethical committee. All patients gave their informed consent to participate.

Six patients received tracer bolus injections of 20 $\mu$Ci 4 $^{14}$C-oestrone and 70 $\mu$Ci of 6,7 $^{3}$H-oestrone sulphate (Table I). The steroids were dissolved in 21 ml saline:ethanol (95/5 w/w) immediately before use. Twenty millilitre was injected. From the residual, 50 $\mu$l samples were obtained for liquid scintillation counting.

Three patients (B.V., S.P. and K.M.) had oestrone sulphate clearance during aminoglutethimide and in a control situation measured in a previous investigation (Lønning *et al.*, 1987). These patients had blood samples obtained on the morning of their steroid injections, from which plasma oestrogens could be measured, and the oestrone sulphate production rate could be calculated.

All patients except S.P., K.M. and L.M. received a bolus injection before and a second injection following 3–21 weeks on aminoglutethimide treatment. The three patients mentioned above were relapsing following 5–8 months on aminoglutethimide treatment. They received a first injection when still on aminoglutethimide therapy, and a second control injection 3–9 weeks following cessation of aminoglutethimide therapy. The one patient (L.M.) who received her secoind injection 9 weeks following withdrawal of aminoglutethimide therapy was then receiving doxorubicin 20 mg weekly (Gundersen *et al.*, 1986).

The steroid injection technique, the blood sampling protocol and the labelled oestrogen analysis were as described previously (Lønning *et al.*, 1987). Blood samples (20 ml) were drawn at 8 a.m. for plasma oestrone and oestrone sulphate determination. The patients then received their tracer oestrogens as a 1 min bolus injection, and heparinised blood samples (10 ml) were drawn through an indwelling needle at 5, 10, 15, 22.5, 30, 45, 60, 90, 120, 150, 180, 210, 240, 300, 360, 480, 600, 720 and 900 min. All samples were centrifuged, plasma separated and stored at −20°C until analysis.

*Oestrogen measurement*

Plasma oestrone, plasma oestrone sulphate and plasma tracer oestrogen concentrations were measured by methods previously described (Lønning *et al.*, 1987, 1989). Samples from each patient were analysed in the same batch. The intra-assay coefficients of variation for radiolabelled oestrone and oestrone sulphate were 6.9% and 8.5% respectively. For plasma oestrogen analysis, intra/inter assay coefficients of variation for oestrone and oestrone sulphate were 8/12% and 7/9% respectively.

*Measurement of aminoglutethimide in serum*

Serum obtained 3 and 12 h following tracer injections were analysed for aminoglutethimide. The mean value of these two determinations were interpreted as a steady state serum level. Aminoglutethimide was determined by the method of Schanche *et al.* (1984).

The three patients (B.V., S.P. and K.M.) who had their oestrone sulphate clearance determined previously were found to have aminoglutethimide plasma levels in the normal range for patients on steady state treatment with that drug schedule (Lønning *et al.*, 1985, 1987).

*Pharmacokinetic calculations*

The metabolic clearance rate of oestrone and oestone sulphate were calculated from the equation:

$$Cl = dose/AUC$$

where dose is the amount of the respective tracer administered and AUC the area under its elimination curve.

In a previous study, we found that oestradiol kinetics could be well fitted to a 3-compartmental model, while the kinetics of oestrone sulphate did not fit into a simple 2- or 3-compartment open model, most probably because of its enterohepatic circulation (Lønning *et al.*, 1987). Therefore, oestrone sulphate AUC was determined by the trapezoidal rule, adding the residual by extrapolation to infinity. The same technique was applied in this investigation. Similar to what has been found by others (Longcope *et al.*, 1974) the oestrone elimination curves in this study were found to be well fitted to a 3-compartmental open model (curve-fitting performed by Statgraphics on an IBM PC 5060). AUC for oestrone was calculated as the integral of the elimination curve. For comparison, it was also calculated by use of the trapezoid rule.

Oestrone and oestrone sulphate production rate (PR) were calculated as:

$$PR = \text{plasma conc} \times Cl \text{ (Gurpide } et\ al.\text{, 1963)}$$

where plasma conc is the endogenous plasma oestrogen concentration before tracer injection, and Cl is the blood clearance rate of the respective oestrogens.

The transfer factors $fm_{Oe_1\text{-}Oe_1S}$ (amount of $^{14}$C-oestrone administered, which appears in plasma as $^{14}$C-oestrone sulphate) and $fm_{Oe_1S\text{-}Oe_1}$ (the amount of $^{3}$H-oestrone sulphate administered which appears in plasma as $^{3}$H-oestrone) was calculated according to the equation (Rowland & Tozer, 1980):

$$fm = \frac{(Cl_{metabolite} \times AUC_{metabolite})}{(Cl_{parent\ compound} \times AUC_{parent\ compound})}$$

The amount of oestrone sulphate produced from circulating oestrone ($Oe_1SPR_{Oe_1}$) was calculated as:

$$Oe_1SPR_{Oe_1} = Oe_1PR \times fm_{Oe_1\text{-}Oe_1S}$$

### Results

The mean ratio between oestrone AUC calculated by the integral and trapezoid methods was $0.98 \pm 0.11$ (s.d.) showing a good agreement. Only the clearance values calculated from the integral AUC are reported.

An example of radioactive plasma oestrogen concent-

**Figure 1** Plasma concentration of $^{14}$C-oestrone (○), $^{14}$C-oestrone sulphate (■) and $^{3}$H-oestrone sulphate (□) in a patient (R.S.) following a bolus injection of tracer oestrogens before commencing on aminoglutethimide treatment.

**Table I** Oestrone plasma concentration ($Oe_1$ conc), oestrone plasma clearance ($Oe_1$ Cl) and oestrone production rate ($Oe_1$ PR) measured in 5 patients before and during aminoglutethimide treatment and in one patient (L.M.) on aminoglutethimide treatment and 6 weeks following cessation of therapy

| | *Control situation* | | | *On aminoglutethimide treatment* | | |
|---|---|---|---|---|---|---|
| *Patient* | *$Oe_1$ conc.* (pM) | *$Oe_1$ Cl* ($l h^{-1}$) | *$Oe_1$ PR* ($nmol h^{-1}$) | *$Oe_1$ conc.* (pM) | *$Oe_1$ Cl* ($l h^{-1}$) | *$Oe_1$ PR* ($nmol h^{-1}$) |
| H.Å. | 133.0 | 51.6 | 6.86 | 80.0 | 63.2 | 5.06 |
| A.L. | 104.0 | 56.3 | 5.86 | 48.0 | 79.6 | 3.82 |
| R.S. | 68.5 | 59.3 | 4.06 | 37.1 | 82.9 | 3.08 |
| S.M. | 139.1 | 61.8 | 8.60 | 55.0 | 66.6 | 3.66 |
| H.S. | 126.3 | 34.2 | 4.32 | 102.7 | 36.3 | 3.73 |
| L.M. | 90.3 | 29.2 | 2.64 | 40.4 | 47.3 | 1.91 |
| Mean | 110.2 | 48.7 | 5.39 | 60.5 | 62.7 | 3.54 |
| Mean % alteration | | | | −45.7% | +29.9% | −30.7% |
| *P* | | | | <0.05 | <0.05 | <0.05 |

**Table II** Oestrone sulphate plasma concentration ($Oe_1S$ conc), oestrone sulphate plasma clearance ($Oe_1S$ Cl) and oestrone sulphate production rate ($Oe_1S$ PR) measured in six patients before and during aminoglutethimide treatment and in three patients (S.P., K.M. and L.M.) on aminoglutethimide treatment and 3–6 weeks following cessation of aminoglutethimide therapy

| | *Control situation* | | | *On aminoglutethimide treatment* | | |
|---|---|---|---|---|---|---|
| *Patient* | *$Oe_1S$ conc.* (pM) | *$Oe_1SCl$* ($l h^{-1}$) | *$Oe_1SPR$* ($nmol h^{-1}$) | *$Oe_1S$ conc.* (pM) | *$Oe_1SCl$* ($l h^{-1}$) | *$Oe_1SPR$* ($nmol h^{-1}$) |
| B.V. | 624 | 6.3 | 3.93 | 185 | 14.5 | 2.68 |
| S.P. | 340 | 6.6 | 2.24 | 118 | 7.9 | 0.94 |
| K.M. | 530 | 4.3 | 2.28 | 195 | 9.4 | 1.83 |
| H.Å | 883 | 4.4 | 3.89 | 330 | 9.1 | 3.01 |
| A.L. | 826 | 6.4 | 5.28 | 154 | 14.2 | 2.20 |
| R.S. | 868 | 3.4 | 2.96 | 184 | 9.5 | 1.74 |
| S.M. | 710 | 7.4 | 5.26 | 240 | 17.8 | 4.28 |
| H.S. | 744 | 6.3 | 4.68 | 232 | 9.3 | 2.16 |
| L.M. | 808 | 2.9 | 2.36 | 120 | 7.0 | 0.84 |
| Mean | 704 | 5.3 | 3.65 | 195 | 11.0 | 2.19 |
| Mean % alteration | | | | −71.3% | +111.8% | −41.0% |
| *P* | | | | <0.005 | <0.005 | <0.005 |

**Table III** Fraction of oestrone metabolised into oestrone sulphate ($fm_1$), fraction of oestrone sulphate metabolised into oestrone ($fm_2$), amount of oestrone sulphate produced by conjugation of circulating oestrone ($E_1SPR^{Oe_1}$) and ratio of total plasma oestrone sulphate calculated to be produced from circulating oestrone ($Oe_1SPR^{Oe_1}/Oe_1PR$) in the same group of patients as described in **Table I**

| | *Control situation* | | | | *On aminoglutethimide treatment* | | | |
|---|---|---|---|---|---|---|---|---|
| *Patient* | *$fm_1$* | *$fm_2$* | *$Oe_1SPR^{Oe_1}$* ($nmol h^{-1}$) | *$\frac{Oe_1SPR^{Oe_1}}{Oe_1SPR}$* | *$fm_1$* | *$fm_2$* | *$Oe_1SPR^{Oe_1}$* ($nmol h^{-1}$) | *$\frac{Oe_1SPR^{Oe_1}}{Oe_1SPR}$* |
| H.Å. | 0.77 | 0.18 | 5.28 | 1.36 | 0.77 | 0.12 | 3.90 | 1.29 |
| A.L. | 0.71 | 0.24 | 4.16 | 0.79 | 0.53 | 0.11 | 2.02 | 0.92 |
| R.S. | 0.91 | 0.45 | 3.69 | 1.25 | 0.76 | 0.21 | 2.28 | 1.31 |
| S.M. | 0.67 | 0.18 | 5.76 | 1.10 | 0.50 | 0.09 | 1.83 | 0.43 |
| H.S. | 0.84 | 0.36 | 3.62 | 0.78 | 0.58 | 0.09 | 2.13 | 0.99 |
| L.M. | 0.82 | 0.38 | 2.16 | 0.92 | 0.83 | 0.20 | 1.59 | 1.89 |
| Mean | 0.79 | 0.30 | 4.11 | 1.03 | 0.66 | 0.14 | 2.29 | 1.14 |
| Mean % alteration | | | | | −16% | −52% | −42% | +15% |
| *P* | | | | | n.s. | <0.05 | <0.05 | n.s. |

rations following a bolus injection is shown in Figure 1 (patient R.S., investigated before aminoglutethimide therapy). Oestrogen disposition parameters in patients during aminoglutethimide treatment and in their control situation are given in Tables I–III. The results are summarised as follows: (1) Aminoglutethimide treatment reduces plasma oestrone and oestrone sulphate levels by a mean of 45.7% and 71.3% respectively ($P<0.05$ and $P<0.005$). (2) The blood production rates of oestrone and oestrone sulphate are consistently reduced by 30.7% and 41% respectively ($P<0.05$ and $P<0.005$). Similarly, the amount of oestrone sulphate produced from oestrone is reduced by a mean of 42% ($P<0.05$). (3) The discrepancy between the reduction in plasma level and production rate for oestrone as well as oestrone sulphate is caused by alterations in the metabolic clearance rates for both oestrogens due to aminoglutethimide treatment. The clearance rate of oestrone is consistently increased by a mean of 29.9% ($P<0.05$), while the clearance rate of oestrone sulphate is increased by a mean of 111.8% ($P<0.005$). (4) A consistent (mean 52%) reduction in the fraction of oestrone sulphate converted to circulating oestrone is seen ($P<0.05$).

The plasma level of aminoglutethimide during steady state treatment was between 3.5 and 12.3 $\mu g\,ml^{-1}$. No correlation between individual plasma aminoglutethimide levels and alterations in oestrogen disposition was seen.

### Discussion

Plasma levels of oestrone and oestrone sulphate were similar to values reported by others (Harris *et al.*, 1984; Roberts *et al.*, 1980; Samojlik *et al.*, 1977; Vermeulen *et al.*, 1978). The clearance and production rates for oestrone in our patients were in the lower range, but consistent with results reported previously for breast cancer patients and normal post-menopausal women (Judd *et al.*, 1982; Kirschner *et al.*, 1978; Longcope & Williams, 1974; Reed *et al.*, 1986). The plasma clearance of oestrone sulphate was similar to values found by us in a previous investigation (Lønning *et al.*, 1987), the oestrone sulphate production rate was found somewhat lower than values reported for young men and menstruating women in the follicular phase of cyclus (Longcope, 1972; Ruder *et al.*, 1972). The transfer constants for oestrone into oestrone sulphate ($fm_{Oe_1\text{-}Oe_1S}$) and oestrone sulphate into oestrone ($fm_{Oe_1S\text{-}Oe_1}$) were in the upper range compared to values reported for younger subjects (Longcope, 1972; Ruder *et al.*, 1972).

The finding that the amount of oestrone sulphate produced from oestrone ($Oe_1SPR_{Oe_1}$) might exceed the total amount of oestrone sulphate produced ($Oe_1SPR$) might be confusing. However, plasma oestrogen levels used for calculating production rate were measured in single samples obtained at 8 a.m. Oestrone sulphate, having a half-life of about 6 h does not show diurnal cycle variations (Noel *et al.*, 1979). One study has suggested a diurnal variation of oestrone in men (Baird & Guevara, 1969). While we have not been able to reproduce this finding in post-menopausal women (Dowsett & Lønning, unpublished results), in some patients a considerable variation in the plasma level during the day may be seen. Therefore, while the oestrone sulphate production rate should be expected to vary little during the day, larger variations in the oestrone plasma levels and therefore the $Oe_1PR$ as well as the $Oe_1SPR_{Oe_1}$ may be anticipated. Our findings support previous results in the literature suggesting that oestrone sulphate is produced by conjugation of circulating oestrone and oestradiol only (Longcope, 1972; Ruder *et al.*, 1972).

In this study we found aminoglutethimide treatment to depress plasma oestrone and oestrone sulphate to about 55% and 30% of their control levels, consistent with previous findings by us (Lønning *et al.*, 1989) and others (Harris *et al.*, 1983; Santen *et al.*, 1982; Vermeulen *et al.*, 1983). The finding that aminoglutethimide treatment causes a 112% increase in the metabolic clearance rate of oestrone sulphate is similar to our previous findings (Lønning *et al.*, 1987).

The clearance rate of oestrone during aminoglutethimide treatment was reported by Santen *et al.* (1978) to be unaffected by aminoglutethimide treatment. In contrast, in this investigation we found oestrone clearance rate to be consistently increased to a moderate extent. Due to a small number of patients in both studies any difference may have occurred by chance. However, it may also be explained by different protocols of investigation. While we used a bolus injection method, Santen and co-workers applied a 4 h steady state infusion technique (Santen *et al.*, 1978). As oestrone sulphate has a half-life about 6 h, there may be doubts whether oestrone steady state levels are reached by such short-term infusions (Hembree *et al.*, 1969). As aminoglutethimide treatment increases the metabolic clearance rate and shortens the half-life of oestrone sulphate, plasma steady state levels for oestrone and oestrone sulphate may be achieved quicker when such infusions are done in patients on aminoglutethimide treatment compared to the control situation. Accordingly, such a flaw could mask a moderate increase in plasma oestrone clearance caused by aminoglutethimide treatment.

An explanation why aminoglutethimide treatment increases the metabolic clearance rate of oestrone sulphate but not oestradiol has been discussed in detail previously (Lønning *et al.*, 1987; Lønning & Kvinnsland, 1988). The discrepancy may be due to a different metabolic clearance rate of the two oestrogens. Oestradiol is a so-called 'highly extracted compound' (Wilkinson & Shand, 1975) with a clearance rate exceeding hepatic plasma flow (Longcope *et al.*, 1968). Therefore, stimulation of its metabolic enzymes may have little impact on oestradiol plasma clearance rate. Oestrone sulphate is a 'low extracted compound' with a clearance rate about 5–10 l $h^{-1}$. If oestrone sulphate metabolising enzymes are stimulated, this may result in an increased plasma clearance rate of this oestrogen. As the clearance rate for oestrone is even higher than the clearance rate for oestradiol (Longcope & Williams, 1974), it may be surprising to find that oestrone clearance rate is increased by aminoglutethimide treatment. However, an explanation may be found in the reduced conversion of oestrone sulphate back to oestrone ($fm_{Oe_1S\text{-}Oe_1}$). While 1–2% of circulating oestrone sulphate will be converted into plasma oestradiol (Ruder *et al.*, 1972), 18–45% of circulating oestrone sulphate was converted into plasma oestrone in our patients. Therefore, a considerable amount of oestrone converted into oestrone sulphate will be reconverted into plasma oestrone. In a previous investigation we found the fraction of oestradiol converted into oestrone sulphate to be reduced by aminoglutethimide treatment (Lønning *et al.*, 1987). In this investigation we found aminoglutethimide to reduce the fraction of oestrone converted into plasma oestrone sulphate as well as the fraction of oestrone sulphate converted into plasma oestrone. These findings are probably due to an increased intracellular metabolism of oestrogens (Lønning & Kvinnsland, 1988). An increased metabolic clearance rate of oestrone sulphate with a reduced conversion of oestrone sulphate to oestrone may result in an increase in plasma oestrone clearance rate.

A moderate reduction in the production rate of oestrone and oestrone sulphate of 30.7% and 41.0% contrasts with previous findings of a 95% inhibition of the aromatase reaction (Santen *et al.*, 1978). However, in recent years the hypothesis that post-menopausal oestrogen production does occur by peripheral aromatisation of androstenedione only has been challenged (Kirschner *et al.*, 1978; Longcope *et al.*, 1982; Reed *et al.*, 1986). Our results indirectly support a theory that oestrogen production pathways other than the aromatisation of androstenedione into oestrone may be intact in patients receiving aminoglutethimide treatment.

The findings in this paper focus on the mechanism of action of aminoglutethimide in the treatment of breast cancer. The successful use of this drug for breast cancer treatment has prompted the development of new aromatase inhibitors undergoing current investigations (Goss *et al.*, 1986). It has been a concept for 10 years that aminoglutethimide depresses plasma oestrogens by inhibiting the peripheral production of oestrone from androstenedione only Santen *et al.*, 1978). Obviously, any other mechanism by which aminoglutethimide may depress plasma oestrogens could be part of its mechanism of action.

Recently, we reported that aminoglutethimide stimulates oestrone sulphate metabolism (Lønning *et al.*, 1987) and reduces the plasma oestrone sulphate/oestrone ratio (Lønning *et al.*, 1989). If plasma oestrone sulphate is an important oestrogen source for breast tumours, an increased metabolism of this oestrogen may be of importance for the mechanism of action of aminoglutethimide. The results of this study suggest that stimulation of oestrone sulphate metabolism may be of equal importance to inhibition of the production rate as a mechanism lowering plasma oestrone sulphate levels. Also, it suggests that the same mechanism may contribute to plasma oestrone depression. Accordingly, our results question aromatase inhibition as the sole mechanism of action of aminoglutethimide.

The findings of this study also suggest that aminoglutethimide treatment does not cause a complete inhibition of post-menopausal oestrogen production, probably due to the existence of alternative production pathways not inhibited by this drug. Further studies on aromatase inhibitors should consider the total production rate of oestrogens as well as aromatase inhibition, to evaluate any possible influence of drug treatment on oestrogen disposition.

This work was supported by grants from The Norwegian Cancer Society. The authors want to thank Mr D. Ekse and Mr A. Høylandskjaer for technical assistance in the oestrogen analysis, and Prof. Per M. Ueland, Mr H. Bergersen and Mrs G. Kvalheim for kindly performing the aminoglutethimide analysis.

**References**

BAIRD, D.T. & GUEVARA, A. (1969). Concentration of unconjugated estrone and oestradiol in peripheral plasma in nonpregnant women throughout the menstrual cycle, castrate and postmenopausal women and in men. *J. Clin. Endocrinol.*, **29**, 149.

DOWSETT, M., HARRIS, A., STUART-HARRIS, R. *et al.* (1985). A comparison of the endocrine effects of low-dose aminoglutethimide with and without hydrocortisone in postmenopausal breast cancer patients, *Br. J. Cancer*, **52**, 525.

GOSS, P.E., POWLES, T.J., DOWSETT, M. *et al.* (1986). Treatment of advanced breast cancer with an aromatase inhibitor, 4-hydroxyandrostenedione: phase II report. *Cancer Res.*, **46**, 4283.

GRODIN, J.M., SIITERI, P.K. & MACDONALD, P.C. (1973). Source of estrogen production in postmenopausal women. *J. Clin. Endocrinol. Metab.*, **36**, 207.

GUNDERSEN, S., KVINNSLAND, S., KLEPP, O., KVALØY, S., LUND, E. & HØST, H. (1986). Weekly adriamycin versus VAC in advanced breast cancer. A randomized trial. *Eur. J. Cancer Clin. Oncol.*, **22**, 1431.

GURPIDE, E., MANN, J. & LIEBERMAN, S. (1963). Analysis of open systems of multiple pools by administration of tracers at a constant rate or as a single dose as illustrated by problems involving steroid hormones. *J. Clin. Endocrinol. Metab.*, **23**, 1155.

HARRIS, A.L., DOWSETT, M., JEFFCOATE, S.L. & SMITH, I.E. (1983). Aminoglutethimide dose and hormone suppression in advanced breast cancer. *Eur. J. Cancer Clin. Oncol.*, **19**, 493.

HARRIS, A.L. DOWSETT, M., SMITH, I.E. & JEFFCOATE, S. (1984). Hydrocortisone alone vs. hydrocortisone plus aminoglutethimide: a comparison of the endocrine effects in postmenopausal breast cancer. *Eur. J. Cancer Clin. Oncol.*, **20**, 463.

HEMBREE, W.C., BARDIN, C.W. & LIPSETT, M.B. (1969). A study of estrogen metabolic clearance rates and transfer factors. *J. Clin. Invest.*, **48**, 1809.

JUDD, H.L., SHAMONKI, I.M., FRUMAR, A.M. & LAGASSE, L.D. (1982). Origin of serum estradiol in postmenopausal women. *Obstet Gynecol.*, **59**, 680.

KIRSCHNER, M.A., COHEN, F.B. & RYAN, C. (1978). Androgen-estrogen production rates in postmenopausal women with breast cancer. *Cancer Res.*, **38**, 4029.

KVINNSLAND, S., LØNNING, P.E. & DAHL, P. (1984). Treatment of breast carcinoma with aminoglutethimide. *Acta Radiol. Oncol.*, **23**, 421.

LONGCOPE, C. (1972). The metabolism of estrone sulfate in normal males. *J. Clin. Endocrinol.*, **34**, 113.

LONGCOPE, C., BOURGET, C. & FLOOD, C. (1982). The production and aromatization of dehydroepiandrosterone in postmenopausal women. *Maturitas*, **4**, 325.

LONGCOPE, C., LAYNE, D.S. & TAIT, J.F. (1968). Metabolic clearance rates and interconversions of estrone and 17$\beta$-estradiol in normal males and females. *J. Clin. Invest.*, **47**, 93.

LONGCOPE, C. & WILLIAMS, K.I.H. (1974). The metabolism of estrogens in normal women after pulse injections of $^3$H-estradiol and $^3$H-estrone. *J. Clin. Endocrinol. Metab.*, **38**, 602.

LØNNING, P.E., JOHANNESSEN, D.C., THORSEN, T. & EKSE, D. (1989). Effects of aminoglutethimide on plasma estrone sulfate not caused by aromatase inhibition. *J. Stewid Biochem.* (in the press).

LØNNING, P.E. & KVINNSLAND, S. (1988). Mechanisms of action of aminogluthethimide as endocrine therapy of breast cancer. *Drugs*, **35**, 685.

LØNNING, P.E., KVINNSLAND, S. & BAKKE, O.M. (1984). Effect of aminoglutethimide on antipyrine, theophylline, and digitoxin disposition in breast cancer. *Clin. Pharm. Ther.*, **36**, 796.

LØNNING, P.R., KVINNSLAND, S., THORSEN, T. & UELAND, P.M. (1987). Alterations in the metabolism of oestrogens during treatment with aminoglutethimide in breast cancer patients. Preliminary findings. *Clin. Pharmacokinet.*, **13**, 393.

LØNNING, P.E. & SKULSTAD, P. (1989). Alterations in the urine excretion of estrogen metabolites in breast cancer women treated with aminoglutethimide. *J. Stewid Biochem.* (in the press).

LØNNING, P.E., UELAND, P.M. & KVINNSLAND, S. (1876). The influence of a graded dose schedule of aminoglutethimide on the disposition of the optical enantiomers of warfarin in patients with breast cancer. *Cancer Chemother. Pharmacol.*, **17**, 177.

NEWSOME, H.H., BROWN, P.W., TERZ, J.J. & LAWRENCE, W. JR. (1978). Medical and surgical adrenalectomy in patients with advanced breast carcinoma. *Cancer*, **39**, 542.

NOEL, C.T., REED, M.J., JACOBS, H.S. & JAMES, V.H.T. (1981). The plasma concentration of oestrone sulphate in postmenopausal women: lack of diurnal variation, effect of ovariectomy, age and weight. *J. Steroid Biochem.*, **14**, 1101.

REED, M.J., BERANEK, P.A., GHILCHIK, M.W. & JAMES, V.H.T. (1986). Estrogen production and nmetabolism in normal postmenopausal women and postmenopausal women with breast or endometrial cancer. *Eur. J. Cancer Clin. Oncol.*, **22**, 1395.

ROBERTS, K.D., ROCHEFORT, J.G., BLEU, G. & CHAPDELAINE, A. (1980). Plasma estrone sulfate levels in postmenopausal women. *Steroids*, **35**, 179.

ROWLAND, M. & TOZER, T.N. (1980). *Clinical Pharmacokinetics: Concepts and Applications*. Lea & Febiger: Philadelphia.

RUDER, H.J., LORIAUX, L. & LIPSETT, M.B. (1972). Estrone sulfate: production rate and metabolism in man. *J. Clin. Invest.*, **51**, 1972.

SAMOJLIK, E., SANTEN, R.J. & WELLS, S. (1977). Adrenal suppression with aminoglutethimide. II. Differential effects of aminoglutethimide on plasma androstenedione and estrogen levels. *J. Clin. Endocrinol. Metab.*, **45**, 480.

SANTEN, R.J. (1986). Aromatase inhibitors for treatment of breast cancer: current concepts and new perspectives. *Breast Cancer Res. Treat.*, **7**, suppl., 23S.

SANTEN, R.J., SANTNER, S., DAVIS, B., VELDHUIS, J., SAMOJLIK, E. & RUBY, E. (1978). Aminoglutethimide inhibits extraglandular estrogen production in postmenopausal women with breast cancer. *J. Clin. Endocrinol. Metab.*, **47**, 1257.

SANTEN, R.J., WORGUL, T.J., LIPTON, A. *et al.* (1982). Aminoglutethimide as a treatment of postmenopausal women with advanced breast cancer. *Ann. Intern. Med.*, **96**, 94.

SANTNER, S.J., FEIL, P.D. & SANTEN, R.J. (1984). *In situ* estrogen production via the estrone sulfatase pathway in breast tumours: relative importance versus the aromatase pathway. *J. Clin. Endocrinol. Metab.*, **59**, 29.

SANTNER, S.J., LESZCZYNSKI, D., WRIGHT, C., MANNI, A., FEIL, D. & SANTEN, R.J. (1986). Estrone sulfate: a potential source of estradiol in human breast cancer tissue. *Breast Cancer Res. Treat.*, **7**, 35.

SCHANCHE, J.S., LØNNING, P.E., UELAND, P.M. & KVINNSLAND, S. (1984). Determination of aminoglutethimide and N-acetylaminoglutethimide in human plasma by reversed-phase liquid chromatography. *Ther. Drug Monitor*, **6**, 221.

THOMPSON, E.A. & SIITERI, P.K. (1974). The involvement of human placental microsomal cytochrome p-450 in aromatization. *J. Biol. Chem.*, **249**, 5373.

VERMEULEN, A., PARIDAENS, R. & HEUSON, J.C. (1983). Effects of aminoglutethimide on adrenal steroid secretion. *Clin. Endocrinol.*, **19**, 673.

VERMEULEN, A. & VERDONCK, L. (1978). Sex hormone concentrations in postmenopausal women. *Clin. Endocrinol.*, **9**, 59.

WILKINSON, G.R. & SHAND, D.G. (1975). Commentary: a physiological approach to hepatic drug clearance. *Clin. Pharm. Ther.*, **18**, 377.